# Implementing a Formalized Risk-Based Approach to Determine Candidacy for Multidisciplinary CKD Care: A Descriptive Cohort Study

**DOI:** 10.1177/20543581231215865

**Published:** 2023-12-01

**Authors:** Maoliosa Donald, Robert G. Weaver, Michelle Smekal, Chandra Thomas, Robert R. Quinn, Braden J. Manns, Marcello Tonelli, Aminu Bello, Tyrone G. Harrison, Navdeep Tangri, Brenda R. Hemmelgarn

**Affiliations:** 1 Department of Community Health Sciences, Cumming School of Medicine, University of Calgary, AB, Canada; 2 Department of Medicine, Cumming School of Medicine, University of Calgary, AB, Canada; 3 Department of Medicine, University of Alberta, Edmonton, Canada; 4 Department of Community Health Sciences, University of Manitoba, Winnipeg, Canada; 5 Department of Medicine, Rady Faculty of Health Sciences, University of Manitoba, Winnipeg, Canada; 6 Faculty of Medicine & Dentistry, University of Alberta, Edmonton, Canada

**Keywords:** chronic kidney disease, kidney failure, kidney failure risk, nondialysis care

## Abstract

**Background:**

The kidney failure risk equation (KFRE) can be used to predict progression to end-stage kidney disease in a clinical setting.

**Objective:**

Evaluate implementation of a formalized risk-based approach in nephrologists’ outpatient clinics and multidisciplinary chronic kidney disease (CKD) clinics to determine candidacy for multidisciplinary care, and the impact of CKD care selection on clinical outcomes.

**Design:**

Population-based descriptive cohort study.

**Setting:**

Alberta Kidney Care South.

**Patients:**

Adults attending or considered for a multidisciplinary CKD clinic between April 1, 2017, and March 31, 2019.

**Measurements:**

*Exposure*—The course of CKD care assigned by the nephrologist: management at multidisciplinary CKD clinic; management by a nephrologist or primary care physician. *Primary Outcome*—CKD progression, defined as commencement of kidney replacement therapy (KRT). *Secondary Outcomes*—Death, emergency department visits, and hospitalizations.

**Methods:**

We linked operational data from the clinics (available until March 31, 2019) with administrative health and laboratory data (available until March 31, 2020). Comparisons among patient groups, courses of care, and clinical settings with negative binomial regression count models and calculated unadjusted and fully adjusted incidence rate ratios. For the all-cause death outcome, we used Cox survival models to calculate unadjusted and fully adjusted hazard ratios.

**Results:**

Of the 1748 patients for whom a KFRE was completed, 1347 (77%) remained in or were admitted to a multidisciplinary CKD clinic, 310 (18%) were managed by a nephrologist only, and 91 (5%) were referred back for management by their primary care physician. There was a much higher kidney failure risk among patients who remained at or were admitted to a multidisciplinary CKD clinic (median 2-year risk of 34.7% compared with 3.6% and 0.8% who remained with a nephrologist or primary care physician, respectively). None of the people managed by their primary care physician alone commenced KRT, while only 2 (0.6%) managed by a nephrologist without multidisciplinary CKD care commenced KRT. The rates of emergency department visits, hospitalizations, and death were lower in those assigned to management outside the multidisciplinary CKD clinics when compared with those managed in the multidisciplinary care setting.

**Limitations:**

The follow-up period may not have been long enough to determine outcomes, and potentially limited generalizability given variability of care in multidisciplinary clinics.

**Conclusions:**

Our findings indicate that a portion of patients can be directed to less resource-intensive care without a higher risk of adverse events.

**Trial registration:**

Not applicable.

## Abrégé

**Contexte:**

L’équation KFRE (Kidney Failure Risk Equation) peut être utilisée en environnement clinique pour prédire le risque d’évolution vers l’insuffisance rénale terminale (IRT).

**Objectif:**

Évaluer la mise en œuvre d’une approche structurée fondée sur le risque dans les cliniques multidisciplinaires d’insuffisance rénale chronique (IRC) et les cliniques ambulatoires des néphrologues afin de déterminer l’aptitude des patients à recevoir des soins multidisciplinaires et de mesurer l’incidence des soins d’IRC reçus sur les résultats cliniques.

**Conception:**

Étude de cohorte populationnelle descriptive.

**Sujets:**

Adultes fréquentant ou envisageant de fréquenter une clinique multidisciplinaire d’IRC entre le 1^er^ avril 2017 et le 31 mars 2019.

**Mesures:**

*Exposition*—le parcours de soins d’IRC attribué par le néphrologue — prise en charge en clinique multidisciplinaire d’IRC; prise en charge par un néphrologue ou un médecin de premier recours. *Principaux résultats*—progression de l’IRC, définie comme l’amorce d’une thérapie de remplacement rénal (TRR). *Résultats secondaires*—décès, visites aux urgences et hospitalisations.

**Méthodologie:**

Nous avons couplé les données opérationnelles des cliniques (disponibles jusqu’au 31 mars 2019) aux données administratives de santé et aux données de laboratoire (disponibles jusqu’au 31 mars 2020). Des modèles de régression binomiale négative et des rapports des taux d’incidence non corrigés et entièrement corrigés ont servi aux comparaisons entre les groupes de patients, les parcours de soins et les environnements cliniques. Les risques relatifs non corrigés et entièrement corrigés de décès toutes causes confondues ont été calculés à l’aide de modèles de survie de Cox.

**Résultats:**

Des 1 748 patients avec une KFRE calculée, 1 347 (77 %) sont restés ou ont été admis dans une clinique multidisciplinaire d’IRC, 310 (18 %) ont été pris en charge par un néphrologue seulement et 91 (5 %) ont été orientés pour une prise en charge par leur médecin de premier recours. Le risque d’insuffisance rénale terminale était beaucoup plus élevé chez les patients restés ou admis dans une clinique multidisciplinaire d’IRC (risque médian à 2 ans : 34,7 %) que chez ceux pris en charge par un néphrologue (3,6 %) et par un médecin de premier recours (0,8 %). Aucun patient pris en charge par un médecin de premier recours n’avait amorcé une TRR; 2 personnes (0,6 %) prises en charge par un néphrologue sans soins multidisciplinaires d’IRC avaient amorcé une TRR. Les taux de visites aux urgences, d’hospitalisations et de décès étaient plus faibles chez les patients pris en charge à l’extérieur des cliniques multidisciplinaires d’IRC comparativement à ceux pris en charge dans ces cliniques.

**Limites:**

La période de suivi n’était peut-être pas été assez longue pour déterminer les résultats. La variabilité des soins dans les cliniques multidisciplinaires pourrait également limiter la généralisation des résultats.

**Conclusion:**

Nos résultats suggèrent qu’une partie des patients pourrait être dirigée vers des soins nécessitant moins de ressources sans hausser le risque d’événements indésirables.

## Introduction

Chronic kidney disease (CKD) is common, affecting approximately 10% of the population,^1^ and is a significant risk factor for adverse outcomes, including morbidity, hospitalization, and cardiovascular events.^2^ Slowing progression of CKD through early detection and management is important for people at all stages of kidney disease.^3^ While identifying people at highest risk of progression is challenging,^4^ tools such as the kidney failure risk equation (KFRE) can be used to predict the risk of progression to end-stage kidney disease (ESKD).^5^ The KFRE uses age, sex, estimated glomerular filtration rate (eGFR), and urine albumin-creatinine ratio (ACR). It has been validated in multiple international cohorts and studied in the context of risk-based triage for nephrology referrals.^6-9^ However, there is limited knowledge regarding implementing the KFRE in outpatient multidisciplinary CKD clinics to support clinical decision-making regarding triage, intensity of care, and the effects on clinical outcomes for people with CKD.

While most people with early stages of CKD can be cared for in the primary care setting,^10^ individuals with advanced CKD are more appropriately managed in a team-based setting that consists of nephrologists, nurses, dietitians, social workers, and pharmacists. These multidisciplinary CKD clinics aim to slow disease progression, manage comorbidities and symptoms, prepare individuals for kidney replacement therapy (KRT), and have been shown to improve health outcomes and provide cost-effective care.^11^ However, there is heterogeneity in multidisciplinary team structure, admission criteria, follow-up, and processes.^12^ For people with CKD, the lack of objective criteria to guide CKD clinic management may lead to a mismatch between the course of CKD care (intensity and complexity of care) that they receive, compared with what is needed.^13,14^

This study is part of a multiphase project evaluating a KFRE-based approach to guide CKD care.^15^ The aim was to evaluate implementation of a formalized risk-based approach in nephrologists’ outpatient clinics and multidisciplinary CKD clinics to determine candidacy for multidisciplinary care, and the impact of CKD care selection on clinical outcomes, including kidney failure, death, emergency department (ED) visits, and hospitalizations.

## Methods

### Risk-Based Approach to Guide CKD Intensity of Care Initiative

Prior to formalizing the KFRE into clinical care (ie, clinical policy), we identified stakeholder perspectives (patients, family members, nurses, nephrologists, and allied health) on potential implementation challenges and solutions. These included the ability for nephrologists to override the KFRE recommendation based on their knowledge of patient characteristics (eg, comorbidities) and nephrologist and patient shared decision-making for referral back to their primary care physician (PCP).^16^ In addition, stakeholders co-created an implementation plan that included a staff communication strategy, implementation support from a research team member, and education and training of nephrologists and staff.

In 2017, the KFRE was integrated into the nephrology electronic medical record (EMR) in nephrologists’ outpatient clinics and multidisciplinary CKD clinics in the Calgary Health Zone, Alberta, Canada, to guide clinician decisions regarding the intensity of care for patients with CKD. The KFRE was applied to patients currently in a multidisciplinary CKD clinic (prevalent patients) at their first routine visit following implementation as well as to new patients being considered for multidisciplinary care by their nephrologists at a routine general nephrology visit (incident patients), with a recommendation for course of CKD care based on their 2-year risk of kidney failure. Only a portion of patients seen by nephrologists in their clinics were considered for multidisciplinary care and, therefore, had a KFRE completed. Incident and prevalent patients at higher risk of kidney failure (2-year risk ≥ 10% or eGFR ≤ 15 mL/min/1.73 m^2^) were recommended to receive multidisciplinary care. Patients with a moderate-risk KFRE (2-year risk 3%-9.9%) were recommended to receive care from general nephrology (remaining under the care of their primary nephrologist). Patients with a low-risk KFRE (2-year risk < 3%) could be referred back to care by their PCP.

### Study Setting and Population

**Figure 1.**
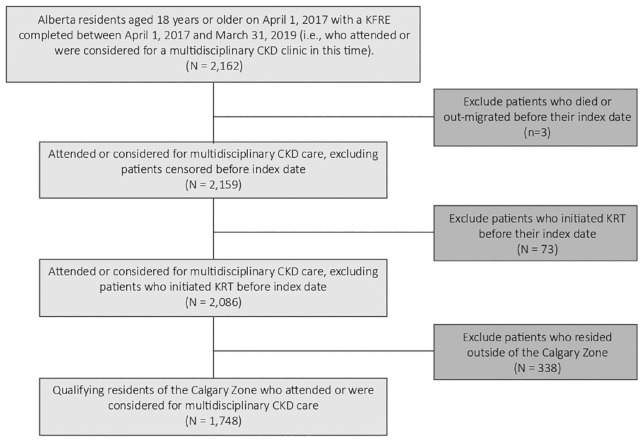
Flow diagram of study main cohort. *Note.* KFRE = kidney failure risk equation; CKD = chronic kidney disease; KRT = kidney replacement therapy.

The multidisciplinary CKD clinic provides team-based care involving nephrologists, nurses, dietitians, pharmacists, and social workers with expertise in CKD care. Patients attending the clinic are seen by their primary nephrologist, along with one or more team members based on their current health care needs. Eligible participants for the main cohort included both prevalent and incident patients. We included adults (≥18 years old) residing in the Calgary Zone who had attended or were considered for a multidisciplinary CKD clinic between April 1, 2017, and March 31, 2019 (Figure 1). We excluded anyone who had commenced KRT prior to the last KFRE assessment and recommendation between April 1, 2017, and March 31, 2019 (ie, the index date). The last recommendation for course of CKD care was most relevant in describing the patient’s disposition during the follow-up period, while we chose the accrual period for those who attended or were considered for a multidisciplinary CKD clinic to maximize the size of that group while having at least 1 year of follow-up data.

### Outcomes and Covariates

We linked operational data from the clinic (available until March 31, 2019) with administrative health and laboratory data from Alberta, Canada (available until March 31, 2020).^17^ Patient consent was not applicable for this study. The primary outcome was CKD progression, defined as commencement of KRT (chronic dialysis or preemptive kidney transplant). Secondary outcomes included all-cause ED visits, hospitalizations, and death. The exposure was the course of CKD care assigned by the nephrologist after the last KFRE assessment between April 1, 2017, and March 31, 2019: remain at or admit to a multidisciplinary CKD clinic; management by a nephrologist (without multidisciplinary CKD care); or management by a PCP. All covariates were defined at the index date. We used patients’ postal codes to include only those residing within the Calgary Zone, an administrative health region which in 2018 comprised 1.6 million people.^18^ We also used postal codes to link with aggregated federal census data to obtain neighborhood household income quintile and urban/rural location. Mean eGFR was calculated from all outpatient serum creatinine measurements in the prior year, using the CKD-EPI equation.^19^ We assessed albuminuria using the most recent urine ACR in the prior year; if an ACR was not available, we used the most recent protein-creatinine ratio (PCR), and if neither were available, we used the most recent urine protein dipstick result. Albumin-creatinine ratio was imputed from PCR and sex using a method developed by our group^20^ and from the dipstick result using a similar method based on same-day ACR and dipstick tests, which also incorporated age, sex, eGFR, and diabetes. We defined comorbidities using validated algorithms based on diagnostic codes in administrative health data^21^ and we calculated 2-year kidney failure risk using the 4-variable KFRE (age, sex, the most recent ACR [measured or imputed], and eGFR).^5^

### External Cohort

**Figure 2.**
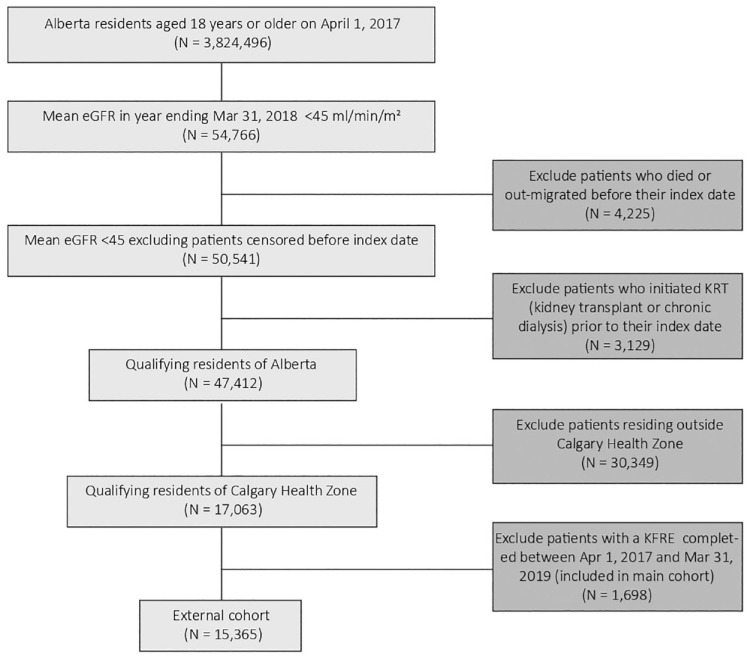
Flow diagram of study external cohort. *Note.* eGFR = estimated glomerular filtration rate; KRT = kidney replacement therapy; KFRE = kidney failure risk equation.

To make a general comparison with patients who were at risk of kidney failure but not managed at a multidisciplinary CKD clinic, we also identified a group of patients who were ≥18 years old, residing in the Calgary Zone, with a mean eGFR <45 mL/min/1.73 m^2^ based on all outpatient serum creatinine measurements in the year ending March 31, 2018, and who did not attend nor were considered for a multidisciplinary CKD clinic between April 1, 2017, and March 31, 2019 (Figure 2). Note that some patients in the external cohort may have attended the clinic before April 1, 2017, or after March 31, 2019. A cut-off eGFR of 45 mL/min/1.73 m^2^ was chosen to include patients who were similar to those being considered for multidisciplinary care. Also, we chose an index date of April 1, 2018, which was the mid-point of possible index dates for the main cohort.

### Statistical Analyses

Initially, we assessed the variation in clinical and demographic characteristics and health service utilization across the 3 courses of care: multidisciplinary CKD care, nephrologist care, and PCP. We also compared the proportion commencing KRT by course of CKD care, category of kidney failure risk, and patient location at the time of KFRE completion (multidisciplinary CKD clinic vs nephrologist’s clinic). In addition, among those who commenced KRT, we compared initial modality and the location of initial KRT by course of CKD care. We compared the ED visit and hospitalization count outcomes by course of CKD care with negative binomial regression count models and calculated unadjusted and fully adjusted incidence rate ratios (IRRs). For the all-cause death outcome, we used Cox survival models to calculate unadjusted and fully adjusted hazard ratios (HRs). All statistical analyses were conducted in Stata 17 (StataCorp, College Station, Texas). Ethical approval to conduct this research was granted by the University of Calgary Conjoint Health Research Ethics Board.

## Results

**Figure 3.**
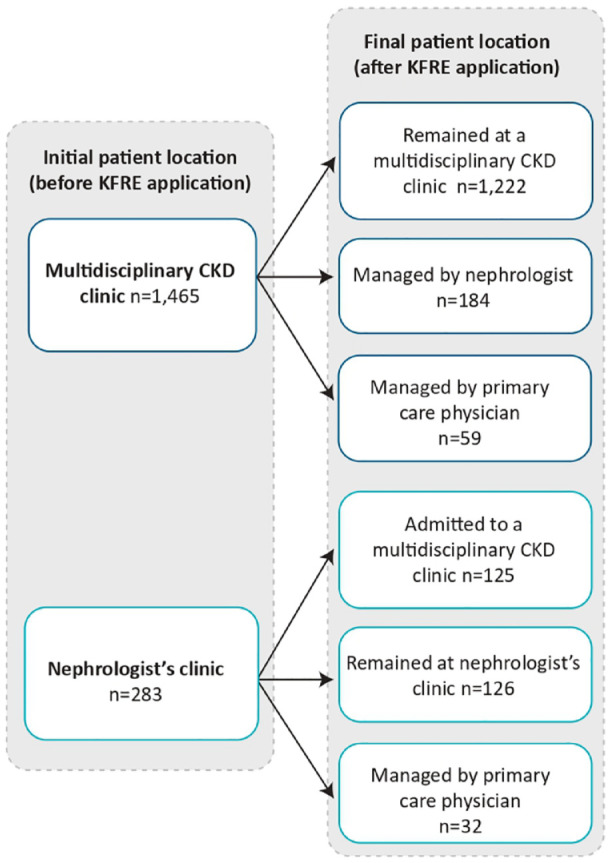
Course of CKD care result after KFRE application. *Note.* CKD = chronic kidney disease; KFRE = kidney failure risk equation.

A total of 1748 patients qualified for the main cohort. Figure 3 shows that of this group, 1465 (83.8%) were attending a multidisciplinary CKD clinic while 283 (16.2%) were being managed by a nephrologist. Table 1 summarizes patient demographic and clinical characteristics by the course of CKD care assigned after application of the KFRE. Patients who remained at (or were admitted to) a multidisciplinary CKD clinic were younger, more likely to be male, had a lower eGFR, and more severe albuminuria than those who were managed by a nephrologist or PCP alone. These characteristics all contributed to a much higher kidney failure risk among patients managed in multidisciplinary CKD care (median 2-year risk of 34.7% compared with 3.6% among those managed by a nephrologist, and 0.8% among those managed by their PCP alone).

**Table 1.** Baseline Clinical and Demographic Characteristics of the Main and External Cohorts.

Characteristic	Main cohort: Course of CKD care (N = 1748)			External cohort N = 15 365
	Multidisciplinary CKD care, N = 1347	Nephrologist, N = 310	PCP, N = 91	
Patient location at the time of KFRE completion, no. (%)				
Multidisciplinary CKD clinic	1222 (90.7)	184 (59.4)	59 (64.8)	—
Outpatient nephrology clinic	125 (9.3)	126 (40.6)	32 (35.2)	
Age (years), median (IQR)	71.0 (60.3, 79.3)	76.3 (68.2, 82.6)	75.6 (65.0, 83.0)	80.8 (72.5, 87.0)
Age category (years)				
<60	24.4	10.6	14.3	6.0
60-69	22.6	21.6	14.3	12.8
70-79	29.8	31.6	31.9	28.6
80-89	19.6	31.6	33.0	38.2
90+	3.6	4.5	6.6	14.4
Male	61.2	58.4	52.8	43.3
eGFR category (most recent measurement, mL/min/1.73 m^2^)^a^				
<15	42.4	0.3	0	1.3
15 to <30	53.4	61.6	19.8	16.5
30 to <45	4.0	31.3	46.2	74.2
45 to <60	0.1	6.1	15.4	7.9
≥60	0.2	0.6	18.7	0.1
eGFR, median (IQR)	16.5 (11.6, 21.1)	27.8 (23.8, 33.3)	38.4 (31.1, 51.8)	38.2 (32.4, 42.3)
Albuminuria: category of measured/imputed ACR (mg/g)				
A1: < 30 (normal/mild)	3.7	34.8	54.9	46.8
A2: 30-300 (moderate)	22.6	46.1	25.3	18.5
A3: >300 (severe)	71.0	18.7	18.7	11.5
No ACR estimate available	2.6	0.3	1.1	23.2
ACR estimate (mg/g), median (IQR)	993 (265, 2431)	51 (18, 183)	27 (8, 126)	19 (13, 115)
Source of ACR estimate				
ACR measurement	93.5	96.1	96.7	37.9
Imputed from PCR and covariates	1.6	1.9	0.0	2.8
Imputed from UDIP and covariates	2.4	1.6	2.2	36.1
No ACR estimate available	2.6	0.3	1.1	23.2
2-year risk of kidney failure (calculated from KFRE, using the most recent lab values prior to index date)				
Low (up to 2.9%)	2.2	44.5	84.6	61.8
Moderate (3%-9.9%)	6.8	47.7	14.3	10.1
High (10% or higher)	90.3	7.7	1.1	4.8
Risk <10% but eGFR < 15	0.7	0.0	0.0	0.1
No KFRE calculation available	0.0	0.0	0.0	23.2
2-year kidney failure risk (%), median (IQR)	34.7 (16.2, 65.9)	3.6 (1.7, 6.1)	0.8 (0.2, 1.6)	0.8 (0.4, 2.2)
Urea, most recent measurement in past 2 years, mmol/L				
<8	0.7	8.1	26.4	18.6
8 to <12	10.5	33.6	29.7	28.0
12 to 16	20.0	28.4	18.7	12.2
>16	64.3	23.6	8.8	7.9
Unmeasured	4.4	6.4	16.5	33.3
Number of outpatient visits with a nephrologist in the past 2 years				
0	1.0	1.6	14.3	75.6
1	3.0	6.8	22.0	7.5
≥2	96.0	91.6	63.7	16.8
Residing in a nursing home or auxiliary hospital at index date	2.2	1.0	2.2	5.3
Rural residence	6.9	3.6	7.7	5.8
Neighborhood income quintile				
1 (lowest quintile)	29.6	24.2	36.3	24.8
2	22.0	21.3	20.9	21.0
3	16.7	19.0	11.0	19.7
4	17	15.5	20.9	17.2
5 (highest quintile)	13.8	19.7	11.0	16.9
Unavailable	1.0	0.3	0.0	0.4
Comorbidities				
Acute myocardial infarction	9.6	12.9	12.1	9.2
Atrial fibrillation	17.9	20.6	20.9	20.7
Dementia	7.3	5.8	9.9	16.6
Diabetes	67.0	55.5	46.2	39.4
Depression	12.4	11.9	18.7	13.0
Heart failure	37.4	37.4	30.8	27.5
Hypertension	94.0	91.9	86.8	90.1
Peripheral arterial disease	12.0	8.1	7.7	7.9
Stroke	25.0	28.7	30.8	25.2

*Note.* Numbers are percentages unless otherwise indicated. CKD = chronic kidney disease; PCP = primary care physician; KFRE = kidney failure risk equation; IQR = interquartile range; eGFR = estimated glomerular filtration rate; ACR = albumin-creatinine ratio; PCR = protein-creatinine ratio; UDIP = urine dipstick/

a Among those not considered for the clinic, eligibility for the cohort was based on mean eGFR in the past year being <45 mL/min/1.73 m^2^. However, some individuals’ most recent eGFR was >45 mL/min/1.73 m^2^.

### Course of CKD Care Versus 2-Year Kidney Failure Risk Category

Among the 1748 patients in the main cohort, nephrologists overrode the tool’s recommended action for 147 (8.4%) (Table 2). In 122 cases (7.0%), patients were managed in multidisciplinary CKD care despite the KFRE tool giving a kidney failure risk of <10% and the eGFR being >15 mL/min/1.73 m^2^. The most frequently provided reason was that the patient had multiple comorbidities which the nephrologist thought could be better managed at a multidisciplinary CKD clinic. In 25 cases (1.4%), patients were managed outside of a multidisciplinary CKD clinic despite the tool calculating a kidney failure risk of >10%. Most frequently, nephrologists either described the patient as stable or noted there had only been one KFRE assessment done.

**Table 2.** Kidney Failure Risk Category and Course of CKD Care (Main Cohort).

Course of CKD care	2-year kidney failure risk estimate				Total
	Recommendation: Management by nephrologist or PCP		Recommendation: Multidisciplinary CKD care		
	<3%	3%-9.9%	≥10%	KFR < 10% but eGFR < 15 mL/min/1.73 m^2^	
PCP	77	13	1^a^	0	91
Nephrologist	138	148	24^a^	0	310
Multidisciplinary CKD care	30^b^	92^b^	1216	9	1347
Total	245	253	1241	9	1748

*Note.* CKD = chronic kidney disease; PCP = primary care physician; KFRE = kidney failure risk equation; eGFR = estimated glomerular filtration rate.

a Indicates patients who were assigned to management by a nephrologist or PCP despite a KFRE-based recommendation for multidisciplinary CKD care.

b Indicates patients who were managed in multidisciplinary CKD care despite a KFRE-based recommendation for management by a nephrologist or PCP.

### Characteristics of People Who Commenced KRT by Course of CKD Care

A total of 360 patients (2.1%) commenced KRT in the year following the index date. Overall, the proportion commencing KRT in 1 year increased with increasing kidney failure risk, from 0.06% among those with low risk (2-year risk < 3%) to 17.4% among those with high risk (2-year risk > 10%) (Table 3). In addition, within the high-risk category, those who were managed in a multidisciplinary CKD clinic had a much higher probability of commencing KRT than other high-risk patients. Similarly, patients who were managed in multidisciplinary CKD care had a much higher probability of commencing KRT (23.5%) compared with those who were managed by their nephrologist or PCP (0.5%). None of the 91 patients managed by their PCP commenced KRT, while only 2 of 310 (0.6%) who were managed by a nephrologist commenced KRT. Among patients receiving care at a multidisciplinary CKD clinic, 0 of 122 commenced KRT with a kidney failure risk of <10%. Meanwhile, 1 of 25 patients who were managed outside of a multidisciplinary CKD clinic commenced KRT with a kidney failure risk of >10%; however, this patient was referred out of the multidisciplinary CKD clinic because dialysis initiation had already been planned. Finally, among patients who commenced KRT, 44% of those who were managed in multidisciplinary CKD care started on peritoneal dialysis (PD).

**Table 3.** Characteristics of Patients Commencing KRT Within 1 Year of Index Date, by Course of CKD Care (Main and External Cohorts).

	Main cohort: Course of CKD care			External cohort	Total
	Multidisciplinary CKD care	Nephrologist	PCP		
Total number of patients	1347	310	91	15 365	17 113
Commenced KRT within 1 year of index date	317 (23.5%)	2 (0.6%)	0 (0.0%)	41 (0.3%)	360 (2.1%)
2-year KF risk category for those who commenced KRT in 1 year (showing percent of those in that risk category)					
Low (<3%)	0/30 (0%)	1/138 (0.7%)	0/77 (0%)	5/9495 (0.05%)	6/9740 (0.06%)
Moderate (3%-10%)	0/92 (0%)	0/148 (0%)	0/13 (0%)	4/1550 (0.3%)	4/1803 (0.2%)
High (>10%)	317/1216 (26.1%)	1/24 (4.2%)	0/1 (0%)	25/735 (3.4%)	343/1976 (17.4%)
Risk <10% but eGFR <15 mL/min/1.73 m^2^	0/9 (0%)	—	—	0/18 (0%)	0/27 (0%)
Risk score unavailable	—	—	—	7/3567 (0.2%)	7/3567 (0.2%)
Initial modality of those who commenced KRT in 1 year					
Hemodialysis	167 (52.7%)	2 (100%)	0	33 (80.5%)	202 (56.1%)
Peritoneal dialysis	140 (44.2%)	0	0	6 (14.6%)	146 (40.6%)
Pre-emptive transplant	10 (3.2%)	0	0	2 (4.9%)	12 (3.3%)
Location of KRT initiation					
Outpatient	202 (63.7%)	1 (50%)	0	14 (34.2%)	217 (60.3%)
Inpatient	115 (36.3%)	1 (50%)	0	27 (65.8%)	143 (39.7%)

*Note.* KRT = kidney replacement therapy; CKD = chronic kidney disease; PCP = primary care physician; KF = kidney failure; eGFR = estimated glomerular filtration rate.

### Prevalent Versus Incident Patients

The 1465 patients who were already attending a multidisciplinary CKD clinic had a higher mean 2-year risk of kidney failure (36.0%) than the 283 who were being considered for admission (17.2%). Correspondingly, 83% of those attending a multidisciplinary CKD clinic remained there, while only 44% of those being considered were admitted. Among those initially attending a multidisciplinary CKD clinic, 297 commenced KRT, of whom 295 remained there; while of those considered for a multidisciplinary CKD clinic, 22 commenced KRT, all of whom were admitted to a clinic.

### All-Cause ED Visit, Hospitalizations, and Death

The unadjusted model for ED visits shows that patients who were assigned to management outside a multidisciplinary CKD clinic (nephrologist or PCP care) had approximately half and two-thirds as many ED visits as those who were managed at the multidisciplinary CKD clinic, respectively (Table 4). Similarly, the unadjusted rate of hospitalizations for both groups was just over half the rate of those who remained at or were admitted to a multidisciplinary CKD clinic. After adjustment for covariates, the differences for both outcomes were attenuated substantially, with only the rate of ED visits among those managed by their nephrologist being significantly lower than those who remained at or were admitted to a multidisciplinary CKD clinic (IRR = 0.79, 95% confidence interval [CI] = 0.63-0.99). Similarly, the unadjusted risk of death was substantially lower among those who were managed outside of the multidisciplinary CKD clinic, compared with those who remained at or were admitted (Table 4). Again, the differences were attenuated by adjustment for covariates, and the adjusted HRs were not statistically significant.

**Table 4.** Models for the Association Between All-Cause Outcomes (ED Visits/Hospitalizations in the Year Following the Index Date, Death) and Course of CKD Care (Main and External Cohorts).

Outcome and course of CKD care	Total no. of patients	N (%) with at least 1 outcome within 1 year	Unadjusted IRR or HR^a^	Adjusted IRR or HR^a^
Outcome: ED visits (IRR)				
Main cohort				
Multidisciplinary CKD care	1347	770 (57.2)	Ref	Ref
Nephrologist	310	138 (44.5)	0.54 (0.43-0.68)	0.79 (0.63-0.99)
PCP	91	44 (48.4)	0.68 (0.47-1.00)	0.92 (0.63-1.35)
External cohort	15 365	5977 (38.9)	0.50 (0.45-0.55)	0.80 (0.70-0.92)
Outcome: Hospitalizations (IRR)				
Main cohort:				
Multidisciplinary CKD care	1347	620 (46.0)	Ref	Ref
Nephrologist	310	96 (31.0)	0.54 (0.41-0.50)	0.91 (0.71-1.18)
PCP	91	24 (26.4)	0.55 (0.36-0.84)	0.84 (0.53-1.33)
External cohort	15 365	4069 (26.5)	0.45 (0.41-0.50)	0.87 (0.75-1.01)
Outcome: Death (HR)				
Main cohort				
Multidisciplinary CKD care	1347	186 (13.8)	Ref	Ref
Nephrologist	310	21 (6.8)	0.56 (0.42-0.76)	0.82 (0.60-1.13)
PCP	91	5 (5.5)	0.36 (0.19-0.68)	0.57 (0.30-1.09)
External cohort	15 365	1310 (8.5)	0.60 (0.53-0.67)	0.77 (0.65-0.92)

*Note.* ED = emergency department; CKD = chronic kidney disease; IRR = incidence rate ratio; HR = hazard ratio; PCP = primary care physician; eGFR = estimated glomerular filtration rate.

a From negative binomial count models (ED visits and hospitalizations) or Cox survival models (death). Models were adjusted for age, sex, eGFR, albuminuria, urea, long-term care residence, rural residence, neighborhood income quintile, and all the comorbidities in Table 1. For the count models, exposure time was censored at death or 1 year after the index date.

### External Cohort

Compared with the main cohort, patients in the external cohort were older and less likely to be male (Table 1). They also had comparable eGFRs and ACRs to those managed by PCPs in the main cohort. These characteristics contributed to a relatively low risk of kidney failure. Consistent with this, a much smaller proportion commenced KRT within 1 year than those who were managed by multidisciplinary CKD care, even among those who were at high risk of kidney failure (Table 3). Among those who did commence KRT, only 15% started on PD, and 66% of dialysis starts were as inpatients.

They also had an unadjusted rate of ED visits and hospitalizations that was about half of those who received multidisciplinary CKD care (Table 4). These differences were reduced substantially in the adjusted models but remained significantly lower for ED visits (IRR = 0.80, 95% CI = 0.70-0.92). Likewise, the unadjusted risk of death was substantially lower among patients in the external cohort compared with those who were managed by multidisciplinary CKD care (Table 4). This was attenuated by adjustment for covariates, but the risk of death was still significantly lower among those not considered for multidisciplinary CKD care (HR = 0.77, 95% CI = 0.65-0.92). Of the 122 people who were managed by multidisciplinary CKD care despite a kidney failure risk of <10%, 13 (11%) died.

## Discussion

The KFRE may be used to guide a risk-based approach for management of patients with CKD^22,23^; however, the wide-spread adoption of clinical decision-support tools into clinical practice has been limited due to having to manually transcribe the KFRE variables into data fields,^24^ and the lack of implementation planning to inform and direct nephrologists in clinical decision-making for patients with CKD. Our risk-based approach initiative integrating the KFRE into clinic EMRs along with a clinical policy within these clinics that clearly outlines the delivery of care defined by KFRE stratification, in addition to allowing for clinicians’ clinical judgment, can enhance the accuracy of the appropriate course of CKD care for patients with CKD. We conducted a population-based descriptive cohort study to evaluate the implementation of this initiative in nephrologists’ outpatient clinics and multidisciplinary CKD clinics to determine candidacy for multidisciplinary care and subsequent impact on clinical outcomes, including kidney failure, death, ED visits, and hospitalizations. Our overall findings indicate that after triaging using the KFRE, 14% of patients were transferred out and managed in less resource-intensive care settings (nephrologist or PCP clinics) and did not result in ESKD or have a higher risk of adverse clinical outcomes.

Multidisciplinary care can provide a holistic approach to care and is relevant for patients who are at high risk of ESKD; however, it is resource-intensive and costly, and may not be globally feasible.^11^ Previous recommendations for multidisciplinary care relied on an eGFR <30 mL/min/1.73 m^2^ as the eligibility entry criterion, but this can be problematic as it has been shown the risk of progression to ESKD is low for a majority of such patients, so they do not require high-intensity management.^25^ Considering patients with a KFRE 2-year risk >10% raises concerns that discharge from multidisciplinary clinics would potentially lead to rapid CKD progression, sooner death, and increased ED visits and hospitalizations. We found that this was not the case for any of these outcomes.

Multidisciplinary care is not warranted for low-risk patients and there is limited evidence demonstrating improvement in health outcomes for these individuals.^26^ Our findings are consistent with prior studies implying that the KFRE is effective at selecting the highest risk subset of patients^25,27^ who benefit from more intensive management. Also, among those who remained at or were admitted to a multidisciplinary CKD clinic, 24% commenced KRT in 1 year, with many starting on PD compared with nonclinic attendees. This is likely related to individuals attending the clinic largely having planned dialysis starts where the multidisciplinary team prepared those at highest risk of kidney failure for dialysis through shared decision-making and education. More importantly, among patients who were reallocated to management by their nephrologist or PCP, only 2 commenced KRT and both had favorable clinical outcomes. Our results are in line with a recent Canadian study finding a lower occurrence of mortality and KRT for patients discharged from multidisciplinary care.^28^

Clinical decision support such as the KFRE can be a useful adjunctive tool for clinical decision-making; however, in certain cases, clinical judgment should be considered. The utility of the nephrologists’ ability to override the recommendation of the KFRE tool based on clinical judgment was a key factor in adopting a risk-based approach to CKD care. In a small number of cases (7%) where the KFRE recommendation was managed outside of a multidisciplinary CKD clinic, nephrologists chose to follow these patients within a multidisciplinary CKD clinic because of their complex needs (ie, multimorbidity).

The results of the study should be considered with its limitations. While we were able to adjust for many clinical factors, there were likely additional characteristics we could not adjust for, raising the possibility of residual confounding. Our follow-up period was 1 year, which may not have been long enough to determine outcomes, especially for low-risk patients. In addition, we lacked data on the course of CKD care after March 31, 2019, so it is possible that some patients were admitted to a multidisciplinary CKD clinic during their outcome ascertainment period. Finally, the care provided by the multidisciplinary CKD clinics may differ from that offered by other multidisciplinary CKD clinics, limiting the generalizability of the study.

In conclusion, planned implementation of a risk-based approach for CKD care supports best practices to guide nephrologists in clinical decision-making regarding the course of CKD care for people with CKD. Our findings indicate that a reasonable number of patients can be directed to less resource-intensive care without a higher risk of adverse events. Future studies evaluating the long-term impact of a point-of-care risk-based approach on patient and health system outcomes are needed.
